# Non-invasively estimated alveolar surface area in extreme preterms: development and respiratory outcomes at two years of age

**DOI:** 10.1038/s41390-024-03527-3

**Published:** 2024-08-25

**Authors:** Emma Williams, Mario Rüdiger, Anusha Arasu, Anne Greenough, Theodore Dassios

**Affiliations:** 1 Women and Children’s Health, School of Life Course & Population Sciences, Faculty of Life Sciences and Medicine, King’s College London, London, UK; 2 Neonatology and Pediatric Critical Care Medicine, Department of Pediatrics, Medizinische Fakultät, Carl Gustav Carus, TU Dresden, Dresden, Germany; 3 Saxony Center for Feto/Neonatal Health, Medizinische Fakultät, TU Dresden, Dresden, Germany; 4 Neonatal Intensive Care Centre, King’s College Hospital NHS Foundation Trust, London, UK

In a recent issue we described a non-invasive method to estimate the alveolar surface area (S_A_) in extremely preterm-born infants by using paired recordings of transcutaneous oxygen saturation (SpO_2_) and fraction of inspired oxygen to calculate the ventilation/perfusion ratio. This was then translated to S_A_ by using Fick’s first law of diffusion and the S_A_ was adjusted with the use of volumetric capnography. We reported that the adjusted S_A_ was lower in infants who required supplemental oxygen at home (home oxygen) compared to those who did not, and was significantly inversely correlated with the duration of inpatient oxygen therapy. We concluded that the adjusted S_A_ described arrested alveolar growth, which is the main chronic pathophysiological phenomenon in premature neonatal respiratory disease, and we proposed the index as a tentative biomarker with potential clinical applicability.^1^

Although the duration of inpatient oxygen and the need for supplemental home oxygen are important, longer-term outcomes such as the total duration of oxygen therapy including the period of supplemental oxygen at home, and growth and development in the early years of life are equally -if not more- important for the affected infants and families.^2^ We aimed, thus, to further test if the adjusted S_A_ as initially measured at seven days after birth was related to the total duration of supplemental oxygen, somatic growth and neurodevelopment assessed at two years of age.

We recorded the total duration of supplemental oxygen therapy in days (including while inpatient and at home), survival to two years corrected age, weight, height, head circumference and neurodevelopmental assessment at two years corrected age. Infants were discharged on home oxygen if at 36 weeks postmenstrual age they required supplemental oxygen via nasal cannulae to achieve an SpO_2_ greater than 93%, they were gaining weight while fully enterally fed and were medically stable.^3^ Infants discharged on home oxygen had an overnight pulse oximetry sleep study within 48 h before discharge and the provided oxygen flow was considered satisfactory if they had a mean SpO_2_ of 93% or above, the SpO_2_ was not below 90% for >5% of the recording period and they did not have frequent desaturation episodes.^3^ The community nursing team and a neonatologist monitored the infants on home oxygen. An overnight pulse oximetry sleep study (heart rate and transcutaneous oxygen saturation) was performed within a week of discharge home and then every two weeks.^4^ The study was considered satisfactory and the oxygen was decreased if the SpO_2_ did not fall below 90% for more than 5% of the recording period, the mean SpO_2_ was 93% or above and there were no frequent desaturation episodes. Weaning was performed in increments of 100 cc/min until a flow of 100 cc/min was achieved, and in increments of 20 cc/min at flows below 100 cc/min. A final sleep study was undertaken once the infant was off supplemental oxygen.

Weight, height and head circumference were measured at follow up and expressed as z-scores.^5^ The Bayley Scales of Infant and Toddler Development, Third Edition (Bayley-III) were administered individually by trained neonatal clinicians. At 24 months corrected age composite scores were recorded in the following domains: motor (gross and fine), cognitive and language (receptive and expressive).^6^ Motor, cognitive or language delay was defined as a composite score of less than 85 in the motor, cognitive or language domain.^7^ The PARCA – R test was utilised if isolation precautions precluded the physical presence of the children in clinic. The composite outcome of moderate or severe neurodevelopment impairment was defined as a delay in any of the motor, cognitive or language domains, an isolated diagnosis of cerebral palsy or global developmental delay.

Data were tested for normality with the Kolmogorov-Smirnov test. They were found to be non-normally distributed and hence were presented as median and range. The relationship of the adjusted S_A_ with the total duration of oxygen was examined using Spearman’s Rho correlation analysis. The adjusted S_A_ was compared in infants with or without moderate to severe neurodevelopmental impairment using Mann-Whitney U non-parametric analysis.

**Fig. 1:**
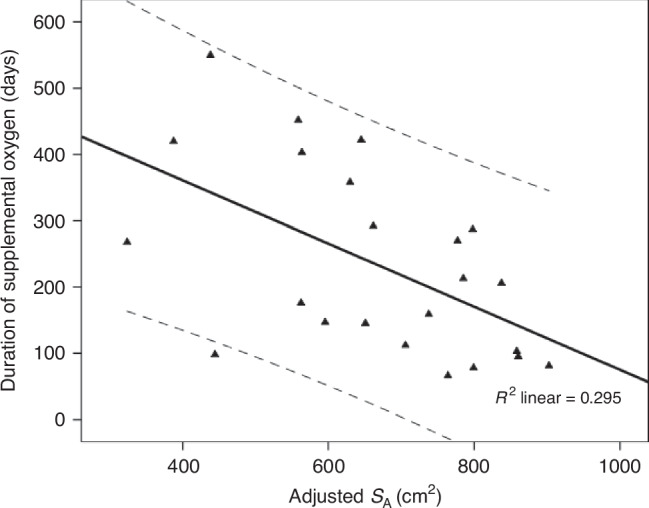
Univariate linear regression of the total duration of supplemental oxygen and the adjusted S_A_. The regression line (solid bold line) and 95% Confidence Intervals (dashed lines) are depicted. R^2^ = 0.295, *p* = 0.007.

Our initial population consisted of 30 infants with a median (range) gestational age of 26.3 (22.9–27.9) weeks and birth weight of 805 (515–1165) gr. The median (range) adjusted S_A_ was 648 (316–903) cm^2^. Two of the infants had died during neonatal care. Of the remaining 28 infants, all were alive at two years corrected age. One infant was still an inpatient and ventilated in paediatric intensive care, due to severe neurological impairment secondary to intraventricular and parenchymal haemorrhage. This infant was excluded from subsequent analysis as the respiratory and non-respiratory morbidity were not thought to be related to extreme prematurity alone. Seventeen infants were discharged on home oxygen and received supplemental oxygen for a median (interquartile - IQR) of 124 (88–174) days (range: 40–550 days). All infants had been weaned off home oxygen by two years corrected age, but two infants were having overnight home non-invasive ventilation without supplemental oxygen. The median (IQR) total duration of oxygen therapy in the 27 included infants was 159 (98–292) days and was significantly related to the adjusted S_A_ (rho = 0.561, *p* < 0.005, Fig. 1).

Follow up at two years of age was performed for 22 of the 27 infants (two declined, one was abroad and two missed the appointment and the parents did not reschedule). The median (IQR) weight [z-score] at follow up was 11.7 (10.6–12.9) kg [z score: 0.15 (−0.78 to 0.61)], height was 83 (80–86) cm [z score: −0.86 (−1.82 to −0.27)] and head circumference was 48 (46.5–48.5) cm [z score: −0.20 (−0.82 to 0.58)]. At follow up, 20 infants underwent a neurodevelopmental assessment, 15 had the Bayley III, two were diagnosed with cerebral palsy or global developmental delay and were unable to complete the assessment, two were reported to have normal development and one had an assessment performed via questionnaire over the phone due to visiting restrictions during the Covid-19 pandemic. For the infants that had a Bayley assessment, the median (IQR) composite cognitive, language and motor scores were 100 (75–105), 79 (68–100) and 91 (82–107) respectively. Ten infants were classified as having moderate to severe developmental impairment and 10 as not. The median (IQR) adjusted S_A_ was significantly lower in the infants with moderate/severe developmental impairment [596 (430–782) cm^2^] compared to infants without moderate/severe impairment [798 (777–859) cm^2^, *p* = 0.04].

We have reported that the adjusted S_A_ as originally measured at seven days after birth was significantly associated with later outcomes such as the total duration of supplemental oxygen therapy and neurodevelopment at two years of age. It is interesting to note that the strength of the association of the adjusted S_A_ with the total duration of oxygen therapy was similar compared to what we had reported for the duration of inpatient oxygen therapy. This observation highlights the early origins of respiratory disease following extreme preterm birth, as events that occurred within the first week of life or during foetal life were sufficient to determine the state of lung health in the first two years after birth.^8^ Our sample of extremely preterm infants was relatively small, but we were able to detect significant differences. We also acknowledge that the neurodevelopmental data were incomplete, but our rate of attendance for the two-year neurodevelopmental appointment (81%) was considerably higher than the one reported at a national (58%) and international level even without travelling and visiting restrictions.^9^

In conclusion, we have reported that the adjusted S_A_ measured at seven days after birth was significantly associated with the total duration of oxygen therapy. We also reported that the adjusted S_A_ was significantly lower in infants with abnormal neurodevelopment at two years of age compared to infants without or with mild impairment at follow up.
